# Ameliorative potentials of Methylcobalamin Vit B12 against teratogenic effects induced by lead in chick embryo

**DOI:** 10.1038/s41598-025-16447-x

**Published:** 2025-10-14

**Authors:** Nazish Ghazanfar, Muhammad Ali Kanwal, Iram Inayat, Syeda Nadia Ahmad, Aima Iram Batool, Waheed Ahmad, Rimsha Zafar, Rabia Idrees, Sadia Suleman, Khawaja Raees Ahmad

**Affiliations:** 1 Department of Zoology, Faculty of Science, University of Sargodha, Sargodha, Pakistan; 2 Department of Zoology, Faculty of Science, University of Chakwal, Chakwal, Pakistan

**Keywords:** Lead toxicity, Methylcobalamin, Chick embryo, Teratogenicity, Heavy metals, Cell biology, Developmental biology, Zoology

## Abstract

Lead (Pb) is a widespread environmental toxicant and potent teratogen known to disrupt embryonic development in various animal models. Methylcobalamin (Vitamin B12), a biologically active form of B12, is reported to exhibit antioxidant and neuroprotective properties. This study aimed to assess the teratogenic effects of lead on chick embryos and evaluate the protective role of methylcobalamin. A total of 200 fertilized golden black chick eggs were randomly divided into four groups: control, lead acetate, methylcobalamin, and lead acetate + methylcobalamin. Eggs were treated on day 0 of incubation and maintained for 14 days. Morphological and morphometric parameters were recorded and statistically analyzed post-incubation. Embryos exposed to lead showed significant growth retardation, reduced body weight and crown–rump length, and multiple morphological anomalies, including microcephaly, limb deformities, and exophthalmos. Co-administration of methylcobalamin markedly reduced the severity of these anomalies and improved growth parameters, indicating a protective effect. The findings demonstrate that lead exposure induces notable teratogenic effects in chick embryos and that methylcobalamin effectively ameliorates these developmental abnormalities. This study supports the potential application of methylcobalamin as a protective agent against heavy metal-induced embryo toxicity in avian models.

## Introduction

Heavy metals are well-known environmental toxicants recognized for their long biological half-lives, persistence, and bioaccumulation potential. They induce oxidative stress, DNA damage, and developmental abnormalities across human and animal models^1–3^.

Lead is a highly toxic heavy metal and a potent teratogen that can cause developmental abnormalities and increased mortality in animals and humans^4^. Because lead is non-biodegradable^5^, it persists in the environment and accumulates in living organisms^6^. Lead exposure can also cause oxidative stress by increasing the production of reactive oxygen species (ROS), which damage tissues during embryonic development^7^. Even low levels of exposure can have harmful effects on health^8^.

Pregnant women with elevated blood lead levels face a higher risk of premature birth and congenital defects^9^. In infants and young children, the blood-brain barrier is particularly sensitive to lead toxicity, which can impair cognitive development^10^. Lead readily crosses the placental barrier and disrupts cellular differentiation, neurogenesis, and vascular formation^11,12^,culminating in severe morphological and functional defects by interfering with critical signaling and gene expression pathways involved in organogenesis^13,14^.

Lead has diverse teratogenic effects across species. Lead-induced teratogenicity is well-documented in avian models like chick embryos, where it disrupts neural tube closure, craniofacial development, and limb formation—primarily through oxidative stress, calcium mimicry, and apoptosis^14–16^. Chick embryo liver treated with lead acetate has shown a shift from normal development to necrotic degeneration^17^. In poultry, broilers exposed to high levels of lead acetate exhibit signs such as weight loss, leg paresis, and skeletal deformities^5^.

Methylcobalamin (MeCbl), the neurologically active form of vitamin B12, has demonstrated potential in mitigating teratogenic effects due to its role in DNA synthesis, methylation, and redox homeostasis^18,19^. Vitamin B12 is an essential micronutrient with antioxidant and neuroprotective properties^20^. It plays a critical role in red blood cell formation^21^ and in maintaining nervous system function^22^.

In previous studies, vitamin B12 supplementation improved growth and development in broiler chickens and reduced the impact of environmental stressors. Methylcobalamin improves neuron function, reducing symptoms of dementia, neuropathic syndrome, Parkinsonism, and Alzheimer’s disease^9,23^. In ovo vitamin B12 supplementation has also shown a positive influence on broiler chicken performance^24^.

Vitamin B12 plays a therapeutic role in suppressing morphological deformities in 14-day-old chick embryos. It significantly increases embryo weight and improves overall body and feather growth^25^. Emerging studies emphasize the protective potential of antioxidants and micronutrients in mitigating such toxic effects during embryogenesis. Recent investigations demonstrate that methylcobalamin supplementation can enhance antioxidant enzyme activities, stabilize mitochondrial integrity^26^, and reduce apoptosis in lead-exposed embryos, leading to improved morphometric and developmental outcomes^27^.

The purpose of this study was to investigate the teratogenic effects of lead exposure on chick embryos and to evaluate whether methylcobalamin (vitamin B12) may mitigate these developmental abnormalities. We hypothesized that vitamin B12 supplementation would reduce the severity of morphological defects and improve growth parameters in embryos exposed to lead.

## Materials and methods

A total of 200 fertilized golden black chick eggs (weight ~ 40 ± 1 g) were collected from the Department of Zoology, University of Sargodha. Eggs were randomly assigned to four experimental groups (*n* = 50 per group):

1. Control group: Injected with 0.1mL distilled water.
2. Lead acetate group: Injected with 0.1mL of 5 mg/kg lead acetate solution.
3. Methylcobalamin group: Injected with 0.1mL of 0.1 mg/kg methylcobalamin solution.
4. Lead + Methylcobalamin group: Single 0.1 ml Injection containing both 5 mg/kg lead acetate and 0.1 mg/kg methylcobalamin.

All the eggs were incubated at 37 °C with 60% humidity.

Dose selection was based on previously reported studies demonstrating teratogenic effects and therapeutic efficacy in avian models^25,26^.

### Dose preparation

Methylcobalamin (vitamin B12) solution (0.1 mg/kg):

Vitamin B12 tablet (Methycobal of 500mcg tablet, a product of Wockhardt Pharmaceutical UK Ltd. having REG No; 016260) was used to prepare 0.1 mg/kg aqueous solution.

### Lead acetate stock solution (5 mg/kg)

For preparation of stock solution Lead acetate was taken.

#### Calculation of lead per gram

1. Stock soln. of lead acetate:5ppm.
2. Mass of lead acetate required = 0.00025 g = 250 µg.
3. Average egg weight = 40 ± 1 g.

Per egg lead acetate = 40/250 = 0.16 µg.

4. Molar mass of lead acetate = 325.29 g/mol.

Molar mass of lead = 207 g/mol.

Mass of lead in lead acetate = 207/325.29 = 0.63 g.

5. Conc. of lead per gram = 0.63 × 0.16 µg = 0.1mL/kg.

### Dose justification

The lead acetate dose used in this study was selected based on previous reports demonstrating teratogenic effects at similar concentrations in chick embryos^16,26,27^.The vitamin B12 dose was adapted, who reported improved hatchability and developmental outcomes in broiler chickens with in ovo vitamin B12 supplementation^23,25^. These doses were chosen to balance observable toxic effects and therapeutic potential while avoiding excessive mortality.

### Dose administration

Eggs were cleaned with a cotton swab soaked in 70% alcohol and maintained in horizontal position for 5–6 min to allow the yolk to rise to the top. Through a micropipette a small drop of concentrated HCL was applied on a small area of egg shell to soften it, as required for safe penetration of needle during dose injection.

Dose was injected using sterilized 1mL syringe into the yolk sac of each egg.

For each treatment group, embryos received a total injection volume of 0.1mL using a calibrated Hamilton microsyringe. In the combined treatment group (Pb + B12), both lead acetate and methylcobalamin were co-administered in a single 0.1mL injection, with adjusted concentrations to ensure that the final delivered doses were 5 mg/kg of lead acetate and 0.1 mg/kg of methylcobalamin, respectively. This was done to maintain uniform injection volumes across all groups and minimize variability due to injection load. The compounds were mixed immediately prior to administration to ensure homogeneity.

Following dose injection, the little window on the egg surface was thoroughly sealed with melted wax. Each egg was weighed using an electric balance.

### Eggs incubation

The eggs were placed into a sanitized HatchPro 56 Egg Incubator, an automated egg incubator with inbuilt candling capacity after dose administration. The incubator was set up for maintaining the temperature at 37 °C and the humidity at 60%. Water was replaced in the incubator after every 48 h during the winter and every 24 h during the summer to maintain normal humidity. Furthermore, the eggs rotation was affixed automatically at every 5 h. The eggs were incubated for 14 days. Regular candling procedure was used in order to examine the eggs thoroughly. Eggs that had no clear signs of embryo development were eliminated.

### Embryos recovery

On 14th day of incubation, embryos were carefully removed from the shell and transferred to 0.9% normal saline solution and subsequently immersed for 48 h in a fixative solution containing 70% alcohol and 30% formaldehyde.

### Morphometry

Each embryo was taken out from the fixative solution and weighed individually using an electric balance. The embryos were kept in a petri dish containing fixative solution to prevent them from drying out. Each embryo was thoroughly examined to identify any morphological deformities. Vernier caliper without zero error was used for morphometric measurements. These measurements were noted for statistical analysis.

### Statistical analysis

The data obtained from chick embryo samples was analyzed by applying ANOVA & ANCOVA test. These tests were carried out by applying IBM SPSS Statistics 23 software.

### Photography and morphological studies

High-resolution images (300 DPI) of all embryos were captured using an 18MP camera in super-macro mode to document and visualize teratomorphogenic anomalies.

## Results

### Morphological results

**Fig. 1:**
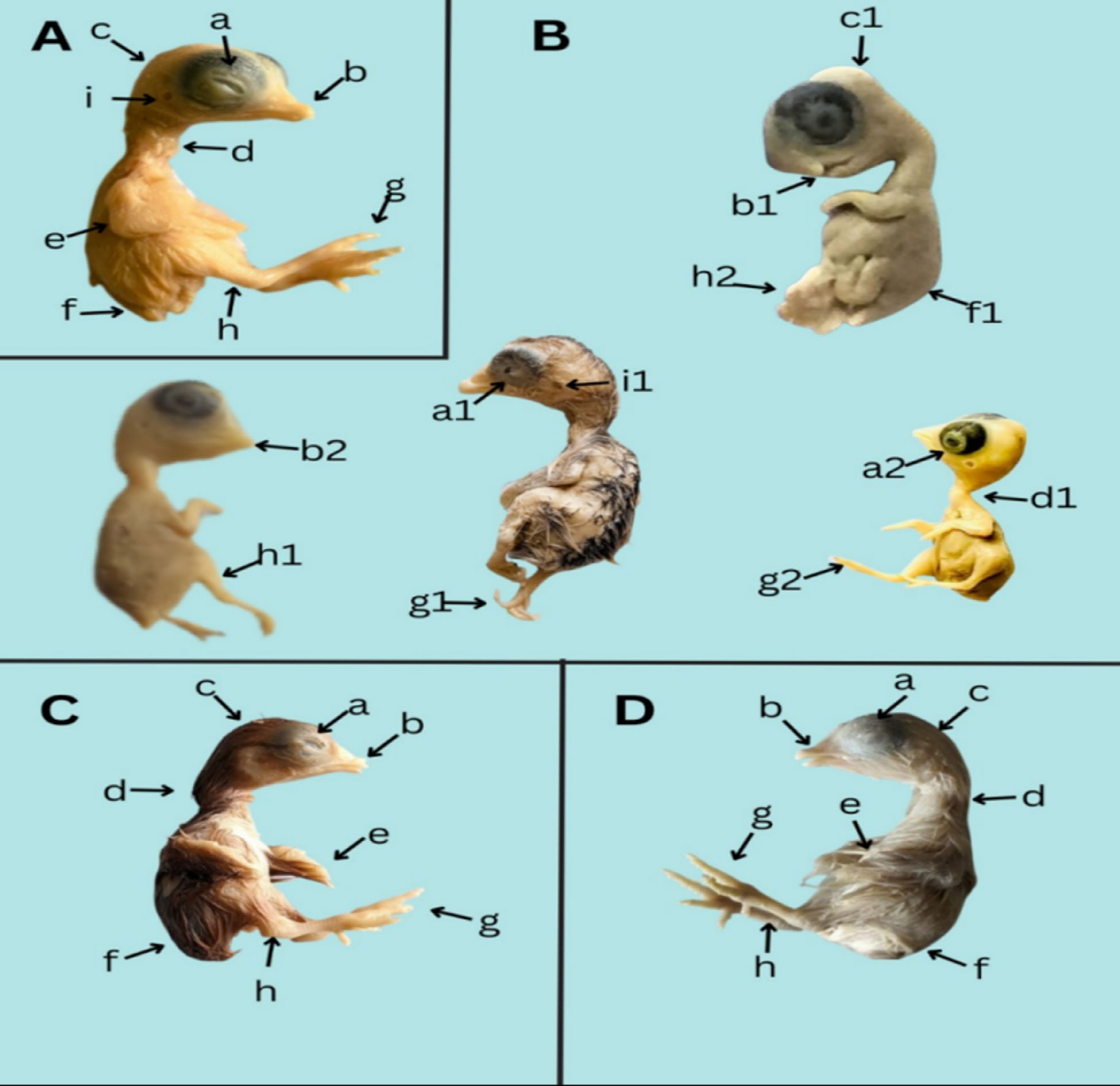
(**a**) Control, (**b**) Pb, (**c**) B12, (**d**) Pb + B12. a: Normal eye development, a1: Permanent open eye having rudimentry eyelid a2: Exophtalmos, b: Normal beak, b1: Rudimentary beak, b2:reduced beak, c: Normal head, c1: microcephaly, d: Normal neck development, d1:skewed neck, e:Normal forelimb, f: Normal down plumage, f1: no down plumage development, g: normal hind limbs development, g1:torted claws development, g2: hind limb with didactyly, h:Normal shank development, h1: reduced shank, h2: Evisceration, i: Normal Auditory meatus, i1: microtia.

The chick embryo in the control group developed normally. All morphological traits such as upper and lower body growth, fore and hind limb development, beak, eye and head growth was normal. Increase in embryo size in 14-days embryo was exactly similar to normal development patterns indicated in the literature (Fig. 1a).

Results revealed different morphological abnormalities in toxin treated group as compare to control group such as significant reduction in embryo weight, forelimb and hind limb, head size, crown-rump length, fronto- occipital length, exophtalmos, eye circumference, beak shortening, skewed neck, microcephaly, micromelia, phocomelia, torted limb especially hind limb, embryonic cataract, muscular dystrophy, reduced beak, tridactyl, rudimentary beak. Furthermore, axial and appendicular growth was also reduced with abnormal growth of phalanges (Fig. 1b).

Vitamin B12 exhibit therapeutic role in mitigating toxic effect induced by lead exposure. Furthermore, Vitamin B12 possess antioxidant role in order to prevent symptoms and manifestations of lead poisoning. Vitamin B12 treated chick embryos shows an elevation of overall development including growth and weight of embryos (Fig. 1c).

Group treated with lead + Vitamin B12 (Pb + B12) shows improvement as compared to toxin group but considerable reduction in size than control group. These embryos showed less severe axial and appendicular growth reduction and muscular dystrophy. Furthermore, recovery of bones and beak size are noted in these embryos. Morphological traits of this group are significantly comparable to control group indicating that Vitamin B12 has potential to mitigate toxic effects induced by lead (Fig. 1d).

### Morphometric results

The mean weight of embryos increased significantly (*p* < 0.05) in the Vit B12 and Pb + B12 groups compared to the Pb-only group. Crown-rump length, biparietal distance, fronto-occipital length, eye width, eye length, eye circumference, shank length, thumb length, and the lengths of index, middle, and little fingers were significantly (*p* < 0.05) decreased in the Pb group but significantly improved in the Vit B12-treated groups. Eye width and eye length showed a significant (*p* < 0.01) increase in the Vit B12 and Pb + B12 groups compared to the Pb group. Upper beak length exhibited a significant (*p* < 0.01) increase in both treatment groups, while lower beak length showed a highly significant (*p* < 0.001) increase in the Vit B12 group compared to the Pb group. Anti-brachium and brachium lengths showed a very highly significant (*p* < 0.0001) decrease in the Pb group, but these measures significantly improved in the Vit B12 and Pb + B12 groups compared to control (Table 1).

**Table 1:** Data table for the statistical evaluations of morphometric parameters variations in 14-day chick embryo morphometric measures.

Morphometric parameters	Mean + SEM			
	Control	Pb	B12	Pb + B12
Mean embryo weight (g)***§	42.16 ± 2.71^a^	35.38 ± 0.94^a^	43.42 ± 1.41^b^	38.49 ± 2.15^a^
Mean crown-ramp length (mm)***§§	55.00 ± 2.33^a^	47.67 ± 0.58^b^	56.07 ± 1.55^a^	52.27 ± 4.13^a^
Mean fronto-occipital length (mm)*§	13.33 ± 2.05^a^	13.02 ± 0.71^ab^	13.92 ± 1.07^a^	13.39 ± 1.63^a^
Mean biparietal distance (mm)*§§	14.05 ± 1.79^a^	10.06 ± 0.61^ab^	14.99 ± 0.93^a^	13.51 ± 1.42^a^
Mean eye width (mm)*§	11.743 ± 1.13^ab^	10.151 ± 0.39^a^	11.844 ± 0.59^a^	10.77 ± 0.90^a^
Mean eye length (mm)*§	9.335 ± 1.14^a^	6.829 ± 0.39^b^	9.939 ± 0.59^a^	8.75 ± 0.90^a^
Mean eye circumference (mm)*§	27.02 ± 8.53^c^	22.03 ± 2.94^b^	28.05 ± 4.45^bc^	26.25 ± 6.76^a^
Mean upper beak length (mm)**§	7.47 ± 1.84^ac^	5.12 ± 0.63^ab^	7.93 ± 0.96^ab^	6.58 ± 1.46^a^
Mean lower beak length (mm)**§	7.265 ± 3.35^a^	5.167 ± 1.16^ab^	7.598 ± 1.75^a^	5.103 ± 2.66^a^
Mean neck length (mm)**§	8.083 ± 2.53^ab^	7.259 ± 0.87^b^	8.758 ± 1.32^a^	8.700 ± 2.01^a^
Mean anti-brachium length (left) (mm)**§	18.91 ± 3.30^abc^	15.52 ± 1.14^b^	18.64 ± 1.72^c^	17.36 ± 2.62^a^
Mean anti-brachium length (right)(mm)***§	9.50 ± 0.34^abc^	6.09 ± 0.51^b^	10.77 ± 0.38^c^	9.04 ± 0.34^a^
Mean brachium length (left) (mm)*§	7.52 ± 1.46^ac^	6.66 ± 0.50^b^	8.20 ± 0.76^c^	7.36 ± 1.16^a^
Mean brachium length (right) (mm)***§	7.68 ± 1.448^b^	5.42 ± 0.500^b^	8.54 ± 0.756^c^	7.25 ± 1.148^a^
Mean whole leg length (mm)*§	29.50 ± 2.37^b^	24.87 ± 0.82^b^	30.56 ± 1.24^c^	26.37 ± 1.88^a^
Mean shank length (left) (mm)*§	13.89 ± 0.452^c^	9.07 ± 0.156^b^	12.14 ± 0.236^a^	11.17 ± 0.358^a^
Mean shank length (right) (mm)*§	11.59 ± 1.16^ac^	7.26 ± 0.40^b^	10.27 ± 0.60^c^	10.26 ± 0.92^a^
Mean thumb length (left) (mm)***§	2.58 ± 0.76^b^	2.59 ± 0.26^b^	3.75 ± 0.39^a^	2.76 ± 0.60^a^
Mean thumb length (right) (mm)** §	4.41 ± 0.46^b^	2.56 ± 0.16^b^	4.69 ± 0.24^c^	3.26 ± 0.36^a^
Mean index finger length (left) (mm)**§	7.48 ± 0.97^ac^	4.65 ± 0.34^b^	6.86 ± 0.51^c^	5.56 ± 0.77^a^
Mean index finger length (right) (mm)*§	7.49 ± 1.00^a^	4.66 ± 0.34^c^	7.76 ± 0.52^b^	6.43 ± 0.79^a^
Mean middle finger length (left) (mm)* §	9.633 ± 1.99^a^	5.796 ± 0.69^b^	10.969 ± 1.04^a^	8.435 ± 1.58^a^
Mean middle finger length (right) (mm) *§	11.33 ± 2.05^c^	7.02 ± 0.71^b^	12.32 ± 1.07^ab^	8.39 ± 1.63^a^

*: *P* ≤ 0.05, **: *P* ≤ 0.001, ***: *P* ≤ 0.0001, §: Analyzed by ANCOVA, §§: Analyzed by ANOV.

## Discussion

Heavy metals such as lead (Pb), mercury (Hg), cadmium (Cd), and arsenic (As) are widely recognized as potent teratogens that impair embryonic development across species. For example, maternal mercury exposure has been linked with intrauterine growth restriction and birth defects in humans^5,27^. Cadmium disrupts skeletal development and organogenesis in avian and mammalian embryos, leading to reduced hatchability and increased mortality^28^. Lead, in particular, accumulates in keratinized tissues and causes long-term toxicity^29^, emphasizing the need for protective strategies like antioxidant therapy.

Recent studies have highlighted the therapeutic potential of vitamins such as B6 and B12 against heavy metal-induced toxicity. Vitamin B6 ameliorated morphological defects in Pb-exposed chick embryos^26^, while methylcobalamin’s effectiveness in reducing developmental toxicity caused by lambda-cyhalothrin is also reported^30^. These findings highlight the broader therapeutic applications of antioxidant vitamins across teratogenic models.

Lead (Pb) remains one of the most toxic and environmentally prevalent heavy metals, known to disrupt embryonic morphogenesis. It readily crosses the placental barrier and interferes with key developmental processes such as neurogenesis, angiogenesis, and skeletal patterning^31^. Avian embryos, such as chicks, have been increasingly used in teratology due to their sensitivity to heavy metals and developmental transparency^32^.Experimental models in chick embryos show that prenatal lead exposure disrupts oxidative balance, interferes with calcium signaling, and causes neural tube defects, craniofacial abnormalities, and impaired organ development^33^.

Oxidative stress plays a central role in Pb-induced teratogenicity. It is characterized by excessive generation of reactive oxygen species (ROS), lipid peroxidation, and mitochondrial dysfunction^32,33^. Methylcobalamin (Vitamin B12) has emerged as a promising therapeutic agent due to its antioxidant properties, DNA synthesis support, and anti-apoptotic mechanisms. It stabilizes mitochondrial membranes, modulates methylation reactions, and protects against oxidative injury by activating antioxidant pathways such as Nrf2/HO-1^34^.

Our study reinforces these observations. We found that Pb exposure significantly reduced embryonic growth, increased morphological deformities (especially in the cephalic region), and led to elevated mortality rates—consistent with previous reports in avian and mammalian models^35,36^. Notably, embryos co-treated with methylcobalamin exhibited recovery in morphometric indices and a reduction in deformities, supporting the compound’s neuroprotective and antioxidant roles.

Lead accumulation in keratinized tissues such as feathers, claws, and beaks was significant and aligns with earlier diagnostic studies^28^. Pb also disrupts neurotransmitter levels, calcium metabolism, and induces apoptosis via epigenetic mechanisms such as aberrant methylation and histone modification^36^. It affects metastable epialleles and alters gene expression, increasing risks of developmental and cognitive disorders^37,38^.

These effects mirror observations in human models where prenatal Pb exposure has been linked with neural tube defects (NTDs), anxiety, and cognitive impairments due to disruption of the blood-brain barrier (BBB)^38^. Even at low concentrations, Pb can induce lasting neurological and behavioral abnormalities in infants and young children^4^.

Rodent models have confirmed that maternal Pb exposure causes significant reductions in fetal body weight, tail length, and crown-rump length, along with elevated neonatal mortality^39^. These teratogenic effects are strongly tied to Pb’s oxidative and epigenetic impact.

In the present study, methylcobalamin demonstrated significant protective effects against Pb-induced teratogenicity in 14-day chick embryos. Embryos treated with methylcobalamin showed higher body weight, improved feather growth, and reduced craniofacial malformations. Similar effects were observed in a study where methylcobalamin alleviated oxyfluorfen-induced toxicity in chick embryos^25^.

Vitamin B12 plays a critical role in maintaining neurological function and redox balance. In ethanol-exposed brain models, it restored oxidant–antioxidant equilibrium, reduced inflammation (via GFAP suppression), and promoted neural growth factors such as BDNF^19^. In another study on rats, methylcobalamin combined with vitamin C significantly countered UV-C-induced oxidative stress, highlighting its free radical scavenging ability^40,41^.

Collectively, these findings confirm that methylcobalamin acts through both direct and indirect antioxidant mechanisms, offering a therapeutic shield against teratogenic agents like Pb. Its potential use in mitigating developmental disorders caused by environmental toxins deserves further exploration in diverse experimental and translational models.

## Conclusion

In this study, lead exposure in chick embryos resulted in pronounced teratogenic effects, including growth inhibition, morphological deformities, and increased mortality. Co-treatment with methylcobalamin significantly reduced these adverse outcomes, improving morphometric parameters and supporting normal development. These findings indicate the potential of methylcobalamin as a protective agent against lead-induced embryo toxicity and highlight its relevance in developmental toxicology.

## Data Availability

Data that support this research work are available for study purposes upon the corresponding author’s justifiable request.
